# Clinical judgement processes of midwives engaged in in-hospital midwife-led births: a qualitative exploratory study

**DOI:** 10.1186/s12884-025-08132-8

**Published:** 2025-10-08

**Authors:** Mami Yamamoto, Aiko Okatsu, Yaeko Kataoka

**Affiliations:** 1 Women’s Health and Midwifery, Graduate School of Nursing Science, St. Luke’s International University, 10-1 Akashi-cho, Chuo-ku, Tokyo, 104-0044 Japan; 2 Maternity Nursing, Faculty of Nursing, Japanese Red Cross Toyota College of Nursing, 12-33 Nanamagari, Hakusan-cho, Toyota, Aichi 471-8565 Japan

**Keywords:** In-hospital midwife-led birth, Clinical judgement, Midwife, Birth, Labouring woman

## Abstract

**Background:**

Japan is facing a significant shortage of obstetricians, particularly in rural areas. To address this problem, the government has been promoting in-hospital midwife-led care to shift certain responsibilities from obstetricians to midwives. The essence of in-hospital midwife-led care lies in the midwives’ judgement and effective practices. This study aimed to understand the clinical judgement process of midwives who provide in-hospital midwife-led births.

**Methods:**

This study employed a qualitative exploratory design using participant observation and think-aloud with nine midwives across three hospitals in Japan. Observations focused on midwives’ clinical behaviours during the first stage of birth. The midwives verbalised their clinical judgements as soon as possible. When they were unable to verbalise, we asked them to describe how they perceived the labouring woman and the type of care or actions they considered engaging in. Data from participant observations and linguistic data gathered during think-aloud were recorded on video. We analysed the data by combining participant observation with clinical judgement using content analysis.

**Results:**

We extracted seven key categories that characterise the clinical judgement processes for midwives during delivery: (1) Predicting the condition of labouring women and determining the initial direction of care upon taking responsibility; (2) Gathering information by asking questions in different ways to identify minor changes in labouring women’s condition; (3) Examining the cause of the problem from available information to uncover and determine the root cause; (4) Predicting birth progress and determining care based on information beyond internal examinations; (5) Instantly modifying the direction of care based on labouring women’s condition; (6) Determining what care should be provided immediately based on predictions of birth progression; and (7) Providing continuous care based on labouring women’s responses to care.

**Conclusions:**

Midwives engaged in in-hospital midwife-led births demonstrated heightened sensitivity to even subtle changes during delivery. They gathered nuanced information through the five senses to predict the progress of birth and provide care. They modified the direction of their care based on the women’s responses and provided the best care for the women on a continuous basis.

## Background

Until the 1950 s, home births were the standard practice in Japan. However, following World War II, under the guidance of the General Headquarters (GHQ) of the Allied Forces, the medicalisation of childbirth began to advance, resulting in substantial promotion of hospital deliveries. Consequently, the number of births occurring in hospitals and clinics increased significantly during the 1960s. Today, most births in Japan occur in medical facilities such as hospitals and clinics [1]. The number of births in Japan has been declining annually, and the total fertility rate fell to 1.20 in 2023 [2]. The total fertility rate in Japan is exceptionally low compared to other countries [3]. This decline can be attributed to several factors, including the increased participation of women in the workforce, the growing number of unmarried individuals, and economic instability. Moreover, the number of obstetricians in Japan has remained stagnant over the past 20 years [4]. This is primarily due to the challenging working conditions in obstetrics, which involve the need for 24-h delivery support and a high litigation risk. The shortage of obstetricians in rural areas is particularly severe [5]. As a result of falling birth rates and a lack of obstetricians, an increasing number of facilities are ceasing childbirth services [6]. Ensuring that women have access to safe and secure environments in which to give birth is an urgent priority. To address this issue, the government is promoting in-hospital midwife-led care for low-risk births managed by midwives rather than by obstetricians [7].

In in-hospital midwife-led care, midwives provide care within medical facilities capable of handling obstetric emergencies. This approach supports the wishes and preferences of the labouring woman and her family while allowing midwives to assess normal and abnormal conditions from pregnancy to approximately one month after childbirth [8, 9]. The proportion of facilities offering in-hospital midwife-led births among all birth facilities nationwide was around 20% in 2023 and has remained unchanged since 2017 [10–12]. Midwifery-led care is increasingly recommended as the first choice for all women, as it has been associated with higher levels of maternal satisfaction and improved birth outcomes [9, 13–16]. In Japan, midwives are authorised to manage only low-risk pregnancies, specifically those that progress normally and are suitable for vaginal birth [17]. To enhance readers’ understanding, we provide information regarding regulations concerning cardiotocography during deliveries in Japan. The obstetrics and gynaecology treatment guidelines in Japan specify the following regarding the use of cardiotocograms: foetal monitoring devices should be used for a minimum of 20 min upon admission to the hospital. If there are no concerns regarding the foetal heartbeat, it is recommended that the device be used again within 6 h. Until subsequent use of the foetal monitoring device, either intermittent foetal heartbeat monitoring every 15–90 min or continuous monitoring is advised [18]. The in-hospital midwifery guidelines state that midwives in charge of in-hospital midwife-led care must assess both normal and abnormal conditions and provide appropriate midwifery care. It is also crucial for them to possess practical skills that allow them to make accurate clinical judgements [8]. Therefore, obstetricians must recognise that midwives are best suited to manage low-risk births [19]. The essence of midwife-led birth lies in the midwife’s judgement and effective practices. The condition of both the woman and the foetus changes rapidly during delivery. Midwives, who closely manage delivery progress, must make accurate judgements regarding whether their condition is normal or abnormal. Expert practitioners often apply knowledge and skills intuitively with no conscious awareness [20]. In the context of in-hospital midwife-led care, midwives provide autonomous and immediate clinical decision making, often drawing on tacit knowledge and experience [21].

No clinical judgement model has surpassed Tanner’s Clinical Judgement Model [22], which has been applied to educational and research settings in the field of nursing [23–25]. Tanner defined clinical judgement as interpreting or integrating a patient’s needs, concerns, or health problems; deciding whether to take action; using or modifying an approach; and improvising what one considers appropriate depending on the patient’s response. This model is applied to clinical situations in which a patient’s condition changes rapidly and requires continuous assessment, reasoning, and response as the situation progresses. This is particularly suitable for describing the clinical judgements of experienced nurses. The clinical judgement model comprises four aspects: noticing, interpreting, responding, and reflection [22]. Noticing refers to the perceptual understanding of a situation and is the ability to grasp the information that nurses need to predict future situations. To understand this situation, nurses must have practical knowledge of patients’ condition and response patterns, knowledge acquired through previous experience, and knowledge gained from textbooks. In addition, the clinical context, including the patient’s condition and environment, influences awareness. Interpreting is the development of an adequate understanding of a situation from the perspective of responding. It allows nurses to understand information and determine appropriate actions. An initial understanding of a clinical situation triggers a pattern of reasoning that leads to interpreting. Responding refers to deciding on a course of action that is considered appropriate for the situation. Interpreting and responding are intertwined; midwives intuitively interpret, react, and corroborate their cognitive patterns. Reflecting refers to paying attention to the patient’s response to midwifery care and reflecting on the nursing interventions practiced. Nurses can make appropriate clinical judgements in similar situations through reflecting. The nurse decides on an action while responding and implement nursing care, leading to the process of reflecting. Midwives make care decisions based on various pieces of information regarding the woman’s condition and subsequently reflect on the care provided in light of the woman’s response.

As Japan’s maternity care system and midwifery-led birth model are unique and may not be familiar to international readers, this background section provides detailed contextual information to foster understanding of the study’s significance.

To date, no research has explicitly described how midwives make clinical judgements during in-hospital midwife-led births in Japan. Therefore, this study aimed to clarify the characteristic clinical judgement processes of midwives who provide this type of care.

## Methods

### Study design and setting

This study employed a qualitative exploratory design using participant observation and think-aloud with nine midwives from three hospitals in Japan [26]. Clinical judgement is triggered by changes in a woman’s condition that prompt midwives to make decisions and implement appropriate care interventions. Midwives’ clinical judgements are informed by information received from labouring women and are closely tied to the specific clinical context. To properly analyse midwives’ clinical judgement process, it is essential to consider not only their clinical judgement but also the situational context in which it occurs. Thus, we chose participant observation and the think-aloud method for our study.

We contacted four facilities that had previously participated in a survey on the promotion of in-hospital midwifery conducted by researchers and selected three that agreed to participate in our study [27–29]. Of the three hospitals, which were located in different regions of Japan, one offered physician-led high-risk births, while the remaining two specialised exclusively in in-hospital midwife-led births. The fieldwork was conducted at each facility for 5–10 days between October 2023 and March 2024.

### Participants and recruitment

The eligibility criteria for midwives included (1) being capable of independently and responsibly managing low-risk births and (2) being in charge of managing women in the first stage of labour during the study period. In our study, we reached out to hospitals that provided midwife-led birth services for their collaboration. Upon obtaining institutional agreement, the head of the maternity department at each hospital identified midwives who met the inclusion criteria. We provided written and verbal explanations of the study to the midwives and obtained written informed consent. Data collection schedules were then arranged in consultation with the midwives. As the study involved observing midwives’ clinical judgement during care provision, obtaining consent from the labouring women receiving care was essential. Researchers explained the following to the labouring women verbally and in writing upon their admission or at the beginning of the midwife’s shift if they were already admitted: (1) the purpose of the study is to observe the midwife’s actions to clarify her clinical judgement, (2) the safety of both the mother and the foetus is the top priority, and there is no risk to their lives, (3) observation will be limited to the first stage of labour, (4) the midwife will make clinical judgements based on the woman’s condition, including her facial expressions and uterine contractions, and these observations will be recorded, (5) internal examinations will not be recorded, and (6) the mother has the right to withdraw from the study at any time. The women’s consent was then obtained.

The sample size was determined using a systematic review of qualitative research sample sizes [30]. We set the number of participants at approximately 10.

### Study procedure and data collection

We observed the midwives providing care to labouring women during the first stage of delivery. The primary focus was on the midwives and labouring women.

To accurately capture the situations in which midwives made clinical judgements, we recorded videos during participant observation. We did not film in areas where the privacy of laborin women could be compromised, such as restrooms or examination areas. In these locations, we recorded only audio. We made every effort to avoid capturing the faces of the labouring women. When family members were present, we obtained permission from both the patient and the family before filming. Additionally, we took care not to film the midwife’s face directly from the front and ensured that we did not interfere with her work. During the filming, we ensured that no identifiable items from the facilities, such as posted notices or personal name tags, were captured. Consent for video recording was obtained from all labouring women, and the video recordings were conducted during participant observation.

Midwives make clinical judgements when they notice changes in labouring women. We asked them to describe how they perceived their patient and what type of care or action they considered taking when they were in charge of her or noticed changes in her condition. In principle, midwives verbalised their clinical judgements (think-aloud) as soon as possible after making them. Midwives engaged in in-hospital midwife-led births often operate in an unconscious competent state, which means that their thoughts may not always be verbally expressed. Midwives’ clinical judgement based on information leads to subsequent care actions. However, due to the intuitive nature of their practice, experienced midwives may have difficulty recalling specific situations or articulating their thought processes if too much time has passed. When it became evident that the midwives struggled to verbally articulate their thoughts and clinical judgements, we encouraged them to think aloud in real-time. During an emergency, the safety of the labouring woman and her foetus is the top priority. In such cases, we asked the midwives to think aloud retrospectively once the situation had stabilised. We asked the midwives to share their thoughts with us outside the delivery room or at the nurses’ station. Data related to the think-aloud task were recorded as audio files.

### Data analysis

During the analysis stage, we carefully restricted access to passwords to ensure that only researchers could view them. We provided detailed descriptions of the labouring women’s condition and the actions of the midwives to ensure that the context of the situation was clear. Linguistic data related to clinical judgements in this context were transcribed verbatim. We reviewed participant observation and clinical judgement data by applying each clinical judgement individually to the elements of context, noticing, interpreting, responding, and reflecting, as outlined in Tanner’s Clinical Judgement Model [22]. This involved deductive content analysis [31]. The participant observation data corresponded to the ‘context’ element of the clinical judgement model. We created codes to represent the characteristics of each extracted clinical judgement process and performed inductive content analysis to categorise and evaluate the codes representing each clinical judgement process. Additionally, we discussed the process of extracting, coding, and categorising each element of clinical judgement.

To indicate that midwives were from the same facility, we assigned them the same letter, and the number following the letter represented individual differences. The next number indicated the number of clinical judgements made. For example, because only midwife A-1 was observed for two days, the first day of observation was labelled A-1-1, and the second day was labelled A-1-2. This numbering system allowed us to distinguish between the two days of observation, with the subsequent number denoting the clinical judgement number.

## Results

The mean age of participating midwives was 41.2 years. All nine individuals had over 5 years of midwifery experience, and two had more than 20 years of professional practice (Table 1).

**Table 1:** Demographics of participating midwives

Midwife’s ID	Facility	Age	Midwifery education background	Years of experience as a midwife	Number of assisted deliveries after obtaining midwifery license	Advanced midwifery certification	Labouring woman’s ID
A-1^*^	A	48	Vocational School	20 years≦	300–399	No	a-1
A-2	A	46	Vocational School	15–19 years	500≦	Yes	a-2
B-3	B	47	Advanced Course (1 year)	15–19 years	100–199	Yes	b-3
C-4	C	45	Advanced Course (1 year)	10–14 years	100–199	Yes	c-4
C-5	C	34	Advanced Course (1 year)	10–14 years	100–199	No	c-5
B-6	B	33	Faculty of Nursing, University	5–9 years	100–199	Yes	b-6
B-7	B	34	Advanced Course (1 year)	5–9 years	100–199	No	b-7^**^
C-8	C	43	Advanced Course (1 year)	20 years≦	500≦	No	c-8
C-9	C	41	Advanced Course (1 year)	10–14 years	200–299	No	c-9^***^

^*^Observed for two days

^**^Was the same labouring woman as b-6

^***^Was the same labouring woman as c-8

We identified seven categories of characteristic clinical judgement processes for midwives engaged in in-hospital midwife-led births during delivery. The seven categories are as follows: ‘Predicting the condition of labouring women and determining the initial direction of care upon taking responsibility’, ‘Gathering information by asking questions in different ways to identify minor changes in labouring women’s condition’, ‘Examining the cause of the problem from available information to uncover and determine the root cause’, ‘Predicting birth progress and determining care based on information beyond internal examinations’, ‘Instantly modifying the direction of care based on labouring women’s condition’, ‘Determining what care should be provided immediately based on predictions of birth progression’, and ‘Providing continuous care based on labouring women’s responses to care’. The category ‘Predicting the condition of labouring women and determining the initial direction of care upon taking responsibility’ consisted of seven codes. The category ‘Gathering information by asking questions in different ways to identify minor changes in labouring women’s condition’ consisted of five codes. The category ‘Examining the cause of the problem from available information to uncover and determine the root cause’ consisted of five codes. The category ‘Predicting birth progress and determining care based on information beyond internal examinations’ consisted of five codes. The category ‘Instantly modifying the direction of care based on labouring women’s condition’ consisted of three codes. The category ‘Determining what care should be provided immediately based on predictions of birth progression’ consisted of three codes. The category ‘Providing continuous care based on labouring women’s responses to care’ consisted of three codes, which were extracted based on consecutive clinical judgements (C-5-2, C-5-3, and C-5-4; Table 2). We provided clear explanations for each category accompanied by code examples, as shown below. Additionally, we indicated the language data related to midwives’ clinical judgements in italics in parentheses. Data that were not language data were referred to as participant observation data.

**Table 2:** The categories of the characteristic clinical judgement processes of midwives engaged in in-hospital midwife-led births during delivery

Category	Code number	Code
Predicting the condition of labouring women and determining the initial direction of care upon taking responsibility	A-2-1	Observing the presence and intensity of contractions to evaluate the woman’s condition at the start of her care
	B-3-1	Considering the risks involved in this birth for this woman
	B-6-1	Considering asking the woman to move around if she is not in active birth when admitted to the hospital
	B-7-1	Performing a palpation and attaching a cardiotocogram to assess contractions at the beginning of care
	C-4-1	Evaluating the woman’s condition at the beginning of care and delivering appropriate care
	C-5-1	Identifying signs of birth progression during hospitalisation
	C-9-1	Trying the crawling position on a woman who is not in active birth
Gathering information by asking questions in different ways to identify minor changes in labouring women’s condition	A-1-1-9	Checking the woman’s bowel movements
	A-1-2-12	Evaluating if the woman is experiencing nausea and monitoring the progress of her birth
	A-2-2	Evaluating the woman’s condition and determining the appropriate care
	B-3-6	Evaluating the effectiveness of contractions and encouraging the woman to go to the toilet
	C-5-2	Carefully assessing the progress of birth without overlooking even minor changes
Examining the cause of the problem based on available information to uncover and determine the root cause	A-2-5	Examining why the intervals between contractions are not decreasing, and suggesting that the woman try hip exercise rotations
	B-6-4	Considering the causes of lower back pain
	B-7-11	Analysing why labour is not progressing and implementing interventions to promote birth
	C-4-3	Exploring the reasons behind the woman’s pain and examining various interventions
	C-5-6	Evaluating and providing care for a woman who is not progressing effectively
Predicting birth progress and determining care based on information beyond internal examinations	A-1-2-9	Assessing anal dilation to confirm the progress of birth
	C-5-7	Returning to the delivery room to ensure a safe delivery as birth progresses
	C-8-3	Estimating the baby’s head position based on anal pressure and suggesting a crawling position
	C-9-5	Evaluating birth progress by utilising external information and attempting a crawling position
	C-9-6	Considering a rotational abnormality, adopting a position that facilitates foetal rotation
Instantly modifying the direction of care based on labouring women’s condition	B-7-2	Integrating findings from internal and external examinations to quickly correct posture
	C-4-2	Performing positional adjustments for bradycardia
	C-9-2	Considering that the labour is progressing because the woman has begun to vocalise
Determining what care should be provided immediately based on predictions of birth progression	A-1-2-7	Allowing the woman to go to the toilet before birth progresses
	C-5-5	Providing care to improve birth progression caused by weak contractions
	C-8-2	Considering and implementing care to improve rotational abnormalities
Providing continuous care based on labouring women’s responses to care	C-5-2	Carefully assessing the progress of birth without overlooking even minor changes
	C-5-3	Detecting changes in the woman and conducting an internal examination
	C-5-4	Considering and providing care strategies that respond to the needs of the woman

### Predicting the condition of labouring women and determining the initial direction of care upon taking responsibility

When midwives first took charge of a labouring woman, they observed her condition, collected missing information, and identified potential risk factors. They predicted the woman’s future condition and determined appropriate care to ensure a smooth delivery. We used the code number B-6-1 to reflect this category below.

#### B-6-1

Labouring woman b-6 was primiparous at 40 weeks and 2 days. She visited the hospital at 6:50 AM. The findings from the internal examination were as follows: cervical dilation of 4 cm, cervical effacement at 60%, and foetal station at −3. Contractions occurred every 7 min, lasting 10–20 s each. The midwife determined that she was not in active contractions and sent her home. At 3:30 pm the same day, the labouring woman returned to the hospital because the contractions became stronger. Findings from the internal examination at this time were as follows: cervical dilation of 8 cm, cervical effacement at 80%, and foetal station at −2. The labouring woman was admitted to the hospital because of the progress of her delivery. After leaving the outpatient clinic, she arrived at the delivery room in a wheelchair. After changing, she lay down on the delivery table, and the midwife attached a cardiotocogram and palpated her contractions.

The midwife recognised that *‘the pregnancy was progressing smoothly’.* She perceived the birth progress: *‘The cervical dilation was 4 cm in the morning and has opened up to 8 cm now, so I think it’s going well’*. However, when she applied the Friedman curve to assess the situation, she found that *‘the interval between contractions was insufficient even though she was in the active phase’.* The midwife intuitively reasoned that the woman had an *‘abnormal rotation’* due to *‘low contractions and severe back pain’.* She felt *that ‘it was necessary to evaluate the progress of delivery using a cardiotocogram and assessing the contraction pain for the woman while also monitoring rotation through internal examinations’.* The midwife observed that *‘the labouring woman is experiencing lower back pain followed by contractions’. ‘The contractions are weak and short despite cervical dilation of 8 cm’.* She suspected that these weak contractions contributed to abnormal rotation.

The midwife predicted early in the woman’s care that she might experience abnormal rotation. This prediction was based on her internal examination findings, the woman’s condition during contractions, and the specific areas of her body experiencing pain. The midwife thought it necessary to evaluate the pain during contractions and perform an internal examination to confirm whether the labouring woman was experiencing abnormal rotation. When the midwife palpated her contractions for the first time, she perceived the duration of the contractions and the order of the areas in which pain from the contractions occurred.

### Gathering information by asking questions in different ways to identify minor changes in labouring women’s condition

The midwives in this study took great care to notice even minor changes in the condition of the labouring women. They adjusted their questioning techniques to assess each woman’s situation accurately. By examining their condition from various perspectives, the midwives gathered a wealth of information that allowed them to make informed decisions to provide the best possible care. Code number A-2-2 was used to explain this category.

#### A-2-2

Labouring woman a-2 was 37 weeks and 6 days multiparous and had given birth twice. Internal examinations were performed by a midwife who was not in charge of this patient. The findings from the internal examination on admission were as follows: cervical dilation of 3–4 cm, cervical effacement at 50%, foetal station at −1, soft cervical consistency, anterior position of the cervix, and unbroken water. The estimated foetal weight was 2,670 g. Cardiotocography showed contraction intervals of more than 5 min. At the nurse’s station, the midwife in charge of the labouring woman observed via the cardiotocogram that *‘the baby’s heartbeat was base 120 bpm, no bradycardia, and reassuring foetal status’.* However, she thought, *‘The cardiotocogram did not pick up contractions well, so I wanted to check the status of the contractions at her side’.* She observed that *‘findings from the internal examination on admission were the same as those during the outpatient internal examination a few days earlier: cervical dilation of 3–4 cm, cervical effacement at 50%, [and] foetal station at −1’.* She believed that because the labouring woman arrived at the hospital experiencing pain from contractions, the delivery could progress based on the intensity and frequency of the contractions. She thought, *‘The birth hasn’t progressed much since the last antenatal care visit, but we need to check her contractions’.* She also made the following prediction: *‘The foetal station is at −1, so if the labouring woman has effective contractions, her delivery may progress faster because the estimated weight of the foetus is approximately 2,600 g’.* As there is no obstetrician in attendance at hospital midwife-led births, *‘the midwife must respond promptly to prevent the transition from normal to abnormal’.* The midwife believed that it was essential to monitor and predict the progress of the birth using her hands, eyes, and other senses rather than relying solely on the cardiotocogram. This approach ensured that she did not miss the slightest changes. Thus, the midwife visited the delivery room to check the actual contraction status by palpation to determine whether the cardiotocogram captured the contractions correctly.

When the midwife entered the delivery room for the first time, the labouring woman looked comfortable and at ease. While palpating the contractions to assess the situation, the midwife asked the labouring woman, *‘Do you feel a push on your anus?’* The woman replied, *‘The anus is…’.* The midwife suspected a minor change due to the woman’s slurring of the words *‘The anus is…’*. On palpation, she noticed that the contractions were weak and asked the labouring woman, *‘You’re having some contractions—do you feel like you want to push?’* The woman responded, *‘Not really’.* The midwife suspected that behind the words *‘Not really’,* the woman might be thinking, *‘I want to push a little’,* suggesting there could be a change for her. To determine what the words meant, she asked the labouring woman, *‘Have there been any changes since your admission?’* The labouring woman responded, *‘I don’t feel like pushing yet so much…’.* The midwife asked her to elicit further information regarding the change in her condition: *‘Do you feel as much pressure on your anus as you did earlier, or do you feel it a little stronger?’* The labouring woman responded, *‘I feel it a bit stronger’.* The midwife wondered ‘*whether “the contractions feel a little stronger” referred to the degree of anal resistance at that moment or if they were getting stronger’.* The midwife reasoned that this might indicate an increased degree of anal resistance. She asked the labouring woman, *‘Is it getting a little stronger?’* The woman said, *‘I feel like my contractions are getting stronger. They were more painful when I was in the car but weakened when I arrived at the hospital’.* The midwife understood that the contractions had become stronger during the drive to the hospital but begun to weaken upon arrival.

The midwife sought to understand the labouring woman’s circumstances by exploring the meaning of her words during the conversation and paying attention to minor changes. Due to the woman’s apparent slurring of words, the midwife altered her questioning approach based on the woman’s responses, aiming to better understand the situation. The midwife’s ongoing effort to understand the woman’s situation was driven by her sense of responsibility and mission to help her continue with a normal midwife-led birth.

### Examining the cause of the problem based on available information to uncover and determine the root cause

The midwives in this study examined the underlying causes of the issues faced by women during labour and identified the root problems. They considered the type of care necessary to address these causes and determined the appropriate course of action. We used the code number B-7-11 to for this category.

#### B-7-11

Labouring woman b-7 was primiparous at 39 weeks and 5 days. She went into labour at 2:00 pm and was admitted at 4:30 pm. The findings from the internal examination on admission were as follows: cervical dilation of 6–7 cm, cervical effacement at 60–80%, and unbroken water. From the time of admission, the labouring woman was positioned supine on her right side, screaming *‘ouch, ouch, ouch’* during contractions and breathing heavily with *‘huffing and puffing’*. The midwife provided anal compression and lumbar massage, encouraging the woman to take deep breaths. She instructed, *‘I’m going to exhale. Huff, huff. Take a big breath in, then huff and puff, and relax’.* The woman’s mother often helped her drink water. At this moment, the midwife perceived that there was *‘not so much anal resistance when touching’.* To determine the cause of her severe pain, she asked, *‘When was the last time you went to the toilet?’* The labouring woman replied, *‘About 4:00 pm?’* The midwife intuitively deduced that it had been approximately 2 h since the woman had last used the toilet. Coupled with the fact that she was frequently drinking water, the midwife suspected that there might have been a buildup of urine in the bladder. This could have been hindering the descent of the foetal head. The midwife said to the woman, *‘I think it might be difficult for you to go to the toilet because the baby may have trouble passing if there is urine in your bladder. Would you like me to insert a tube for urinary drainage?’* She then proceeded with the procedure. The urine was concentrated, and the volume was 20 mL. At that moment, the midwife considered the possibility of a foetal rotation issue. This concern arose* ‘because the woman was experiencing intense pain, and although contractions were occurring approximately every 3 min, there was no anal resistance. Additionally, early decelerations were noted on the cardiotocogram’.* The midwife decided to perform an internal examination to assess foetal rotation while conducting urinary drainage. The findings from the internal examination were as follows: cervical dilation of 9 cm, cervical effacement at 90%, foetal station at −3, and the anterior fontanelle positioned at 6 o’clock. The midwife told the woman, *‘The delivery is progressing very well. Currently, the cervical dilation is 9 cm and almost fully open. However, the foetus may be larger than expected and descending slowly’.* At that moment, the midwife reflected, *‘The foetal position is at −3, and the estimated foetal weight is large, which means it will take some time to deliver the baby. The woman is becoming restless in her side-lying position, so I need to find a more comfortable position to help her relax’.* She told the labouring woman, *‘Lying on your side doesn’t put much pressure on your legs, so it might be easier for you to get on all fours. This position can help the foetus turn more easily, and it will allow your legs to rest on the delivery table more comfortably. If you try it and find that lying on your side is better, we can return to the original position’.* She then assisted the labouring woman into the crawling position.

In this situation, the midwife analysed the reasons for the woman’s severe pain, ruling out each possible cause individually. Ultimately, she concluded that the foetus had descended slowly owing to its estimated large weight. She consistently made decisions and provided care while considering the woman: how to facilitate the birth’s progress and which positions could help the woman feel calmer during contractions. The infant was born weighing 3,590 g, which is heavy for the gestational age, and the birth took approximately 3 h to occur. The midwife predicted a slow descent of the foetal head owing to its size, having ruled out other obvious factors.

### Predicting birth progress and determining care based on information beyond internal examinations

Frequent internal examinations can be painful for women. To minimise discomfort, the midwives in this study strived to avoid unnecessary internal examinations and instead relied on external examinations to monitor the birth progress. When the midwives determined through external examinations that the birth was not progressing smoothly, they considered and implemented care strategies to facilitate a smoother birth. We used the code number C-9-6 for this category.

#### C-9-6

Labouring woman c-9 was multiparous at 39 weeks and 5 days and had given birth once. She was admitted to the hospital at 6:00 am due to contractions. Findings from the internal examination at 9:00 am remained unchanged from admission: cervical dilation measured 7 cm, and foetal rotation remained unclear.

This scene occurred at 12:40 pm. The midwife had not conducted any internal examinations since 9:00 am. The midwife assessed the labouring woman’s condition: *‘The contractions are distant. They have lessened in strength, now occurring every 4–5 min’.* The labouring woman ate lunch while sitting. The midwife told her, *‘I will conduct one internal exam before placing the cardiotocogram on you’.* The woman asked the midwife, *‘May I use the toilet?’* The midwife escorted her to the toilet. The midwife noticed that the woman was able to eat 80% of her lunch. She also observed that *‘the contractions were either infrequent or weak’.* Based on her observations, the midwife intuitively reasoned that it was safe for the woman to use the toilet because delivery was not currently in progress. The midwife accompanied the woman to the toilet. After returning from the toilet, she glanced at the pad and noticed that the woman’s water had broken as she was preparing to perform an internal examination. She then asked the woman, *‘Did your water break?’* The woman answered, *‘I don’t know’.* The midwife first confirmed that the water had broken using an amnicator test and then conducted an internal examination. The colour of the amnicator swab changed from yellow to blue. The midwife informed the woman during the internal examination that *‘cervical dilation is at 7 cm, cervical effacement is thick, and the foetal station is −2. There does not appear to be any cervical effacement. The water has broken’.* She was uncertain and lacked clarity regarding the foetal sagittal suture. At that moment, the midwife noticed that *‘the foetal heartbeat is unclear. I suspect that the foetal back is positioned against the woman’s back. If the foetal back were turned toward the woman’s front, I believe I would be able to hear the heartbeat more clearly’.* She intuitively reasoned that the foetal position might be in the right occiput posterior (ROP) based on the unclear sound of the foetal heartbeat. She thought that if the foetus was in ROP, it would be more likely to turn if placed in the left lateral position. She advised the woman, *‘Since you are in ROP, please turn to your left’,* and the woman was placed in the left lateral position. The midwife hoped for the foetus to turn as the woman was placed in the left lateral position.

In this situation, the midwife predicted the progress of birth based on information other than internal examination at two key points. First, she determined that the birth was not progressing because the woman was able to consume 80% of her lunch while sitting up; additionally, she noted the status of the woman’s contractions. Second, although the midwife could not identify the sagittal suture during the internal examination, she was able to predict the foetal rotation based on an unclear heartbeat. The midwife assessed the woman’s situation through various observations and internal examinations and selected the most appropriate care.

### Instantly modifying the direction of care based on labouring women’s condition

The midwives in this study swiftly evaluated the women’s condition and adjusted their care based on the assessment. We used the code number B-7-2 for this category.

#### B-7-2

Labouring woman b-7 was primiparous at 40 weeks and three days. Internal examination revealed a cervical dilation of 10 cm, cervical effacement at 100%, foetal station at −1, and posterior fontanelle positioned at 10 o’clock. After the internal examination, the labouring woman was placed in the left lateral position. The midwife gently massaged the woman’s lower back when contractions occurred. Once the contractions eased, she instructed the woman to lie on her back. After performing Leopold manoeuvres, she attached a cardiotocogram to monitor the foetal heartbeat and contractions. The woman positioned herself lying on the right side.

In this situation, the midwife said, *‘The foetal head is not coming perpendicular to the pelvic inlet plane’.* She did not overlook trivial information. The midwife conducted Leopold manoeuvres and noted that *‘the foetal back is on the right side of the woman’s body’.* The midwife intuitively reasoned from her experience, *‘Gravity helps the foetus turn more easily when its back is positioned on the ventral (anterior) side of the woman during stronger contractions’.* The woman had just rapidly changed positions from a left lateral position to a supine position to a right lateral position. However, the midwife decided that the left lateral position would better encourage foetal rotation. The woman first needed to lie down in the supine position in order to then allow her to move from the right to the left lateral position. This change in position leads to contractions and discomfort. The midwife thought, *‘If the woman does not mind, she might be willing to change to the left lateral position’.* The midwife observed the woman to determine if she was willing to change positions. The midwife intuitively sensed that *‘the woman was going to do it’.* The midwife told her, *‘The foetus is on your right side, so it may be easier to turn if you change your position to the left’.* Following this advice, the woman switched from lying on her right side to lying on her left.

It took approximately 2–3 min after the internal examination to explain to the woman how to change her position to the left lateral position. During this 2- to 3-min period, the midwife quickly became aware of the status of foetal rotation based on her internal examination findings and information gathered from the Leopold manoeuvres. The foetus is more likely to turn if the woman is in the side-lying position than in the supine position. To facilitate foetal rotation, it is better to lie on the side with the foetus on top. The midwife thought this immediately and changed the woman’s position from right to left.

### Determining what care should be provided immediately based on predictions of birth progression

The midwives in this study assessed the mothers’ current condition to predict how their situations might evolve. They considered what care could be provided at that moment to prevent any complications during delivery and took appropriate action. They consistently provided care with foresight. We used the code number C-8-2 for this category.

#### C-8-2

Labouring woman c-8 was 39 weeks and 5 days multiparous and had given birth once. Cervical dilation was at 7 cm, with the anterior fontanelle at 2 o’clock. The midwife confirmed the foetal rotation with an abdominal ultrasound, which showed the foetal face facing upward. She observed that *‘the rotation is not optimal because the anterior fontanelle is positioned at 2 o’clock, and the foetal face is facing upward’.* She predicted that *‘since the woman is a multipara and her cervical dilation is at 7 cm, the foetal rotation appears good, so the foetus should have been born by now. However, because the rotation is not optimal, the foetus will take a little longer to deliver’.* The midwife explained, *‘The back of the foetal head should be positioned on the side of the woman’s abdomen. Then, the foetus will come out easily’.* She intuitively reasoned that the birth could progress more quickly if foetal rotation returned to normal. She thought, *‘The more the woman moves, the more the foetus will turn around’,* which would facilitate the delivery. In addition, the woman had not used the toilet since admission. The midwife thought, *‘Urine retention in the bladder will be a factor in preventing the foetal descent’.* Urine accumulates over time, and contractions are intensified by movement. Therefore, the midwife believed that it would be better for the woman to use the toilet at that time, considering the future progress of the birth. The midwife concluded that ‘*the cervical dilation is open, but the foetal head is positioned high, and the woman is unable to push naturally. She is having contractions every 2–4 min but does not feel any pain. She can go to the toilet now; this is the right time for her*’.

In this case, the midwife recognised the abnormal rotation through internal examination and abdominal ultrasound and anticipated the risk of prolonged delivery if the rotation did not normalise. On the other hand, she was also trying to determine what care should be given now to return the foetus to normal rotation while anticipating what course the labouring woman might take in the future.

### Providing continuous care based on labouring women’s responses to care

**Fig. 1:**
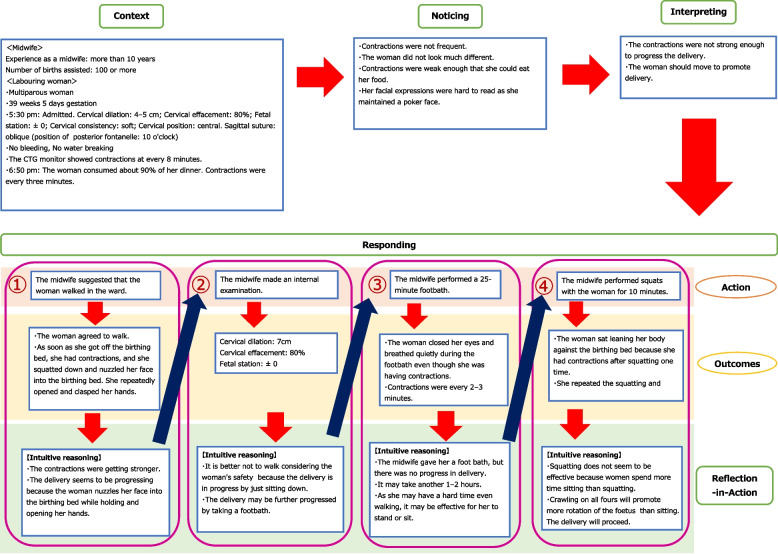
Category structure: providing continuous care based on labouring women’s responses to care

The midwives in this study noticed the slightest changes in the women they cared for as they were continuously involved in their experiences. Their primary goal was to guide the women toward a natural birth by providing the best possible care. They also made prompt inferences based on the women’s responses to their care, adjusting their approach as needed to ensure continuous support and optimal care throughout the process. We used the code numbers C-5-2, C-5-3, and C-5-4 for this category (Fig. 1).

#### C-5-2, C-5-3, and C-5-4

Labouring woman c-5 was multiparous at 39 weeks and 5 days and had given birth twice. She was admitted at 5:30 pm. Upon admission, an internal examination revealed the following findings: cervical dilation of 4–5 cm with 80% cervical effacement. The foetal station was noted at ± 0, the cervical consistency was soft, and the position of the cervix was central. Additionally, the posterior fontanelle was positioned at 10 o’clock. Contractions were occurring every 8 min.

One h had elapsed since admission. The midwife visited the room while the woman was having dinner and found that *‘she is feeling much the same and eating well’.* Based on her previous experience, she thought, *‘The woman wouldn’t be able to eat if she felt pain’.* However, because the woman managed to eat almost 90% of her food, the midwife concluded that *‘the contractions must not be powerful’.* She asked, *‘Are your contractions strong or weak?’* The woman nodded in response. The midwife then asked her if she felt like straining. She replied, *‘Not yet’.* She asked the midwife, *‘Is it okay for me to move even if it hurts?’* The midwife thought, *‘The woman should move slightly to speed up the birth’.* She told the woman, *‘The birth would likely go faster if you moved’.* She then thought, *‘The woman should be fine walking, as she doesn’t appear to be in much pain’.* On the other hand, she reflected that *‘since the woman is multipara, her condition could change rapidly. We need to be cautious about her. If we notice any changes during the external examination, we should conduct further internal examination and reassess her condition’.* She further thought, *‘The woman has a poker face and is difficult to read’.*

The woman had just gotten off the delivery table when a contraction hit her. She leaned against the table and squatted. At this point, the midwife thought, *‘The woman squatted down when she tried to move, so I guess she feels a lot of pain. I wonder if the birth is progressing’.* The midwife thought, *‘Let’s conduct an internal examination since she squatted. We must evaluate whether she can walk or leave the delivery room’.* She decided to perform an internal examination before allowing the woman to walk. She conducted an internal examination, which revealed that the cervical dilation measured 7 cm, with 80% cervical effacement, and the foetal station was at ± 0. Based on this internal examination, she believed that *‘the birth was progressing while seated’.* She believed that *‘the woman’s birth may progress even with a footbath’.* She told the woman, *‘Let’s sit down and take a footbath’.* She thought, *‘If the contractions become stronger, the woman can move to the delivery table immediately’.* Therefore, she decided to give the woman a footbath in the delivery room instead of having her walk around the ward.

The midwife gave the woman a 25-min footbath. The woman looked down and breathed quietly during her contractions, and the midwife gently palpated them to confirm their strength. The contractions occurred every 2–3 min, lasted 10 s each. The midwife noted, *‘The woman does not feel the strong urge to push. She doesn’t sense the foetal head pressing against her buttocks, which means each contraction is weak. The progress of the birth relies on the strength of the contractions’.* She informed the woman, *‘I can’t say for certain because you have given birth before, but it may take 1 to 2 h to deliver the baby’.* At this point, the midwife thought, *‘I don’t believe the birth is progressing well. The contractions are not very strong. To help facilitate the birth, you need to walk a little more’.* She told the woman, *‘Let’s finish the footbath, and then we can walk away’.* The woman nodded. The midwife completed the footbath by gently drying the woman’s feet. She observed that *‘the woman would likely have difficulty walking, as she had been looking down with her eyes closed and breathing deeply and quietly through the contractions’.* She concluded that *‘performing squats could be beneficial, as standing or sitting up would help advance the progress of the birth’.*

The midwife advised the woman, *‘You may stand or sit here while holding on to something’.* The woman squatted while holding onto the delivery table, but after one squat, a contraction hit her, and she sat on the floor, resting her body on the delivery table. When the contractions subsided, she resumed squatting; however, the contractions soon returned, and she sat up again. This process was repeated five times. The midwife thought, *‘Squatting is ineffective because the birth didn’t progress quickly, and the woman would sit up and get stuck when she squatted’.* The midwife asked the woman to get down on all fours to encourage foetal rotation.

A unique feature in this theme was that the process of ‘responding’ to ‘reflecting’ was continuously conducted four times. During this process, the midwife collected extensive information from the woman and observed her condition from various perspectives. She developed various reasons while perceiving the woman’s condition and selected the best care for her. She provided all possible care within the limited space of the delivery room.

## Discussion

In this study, the midwives who provided in-hospital midwife-led births improved their understanding of labouring women by asking questions and observing them closely. This approach enabled them to make accurate judgements. They drew various conclusions from the information gathered to determine the best care for each woman. Additionally, the midwives considered and adjusted their interventions based on the women’s responses to the care they provided.

### A deeper understanding of labouring women through careful questioning and continuous observations

Understanding patients is crucial to delivering high-quality, individualised care [32, 33]. Midwives working in in-hospital midwife-led units observed a range of changes in the women under their care. Their ability to detect subtle variations in women’s condition stems from their continuous and intimate involvement in the delivery process [34]. Another critical piece of information is language. However, the levels of expression and perception vary from person to person. To properly understand the women, the midwives needed to ensure that they accurately grasped the intent behind the women’s words. They aimed to comprehend the meaning of their nuanced expressions by asking the women questions for clarification whenever necessary. Critical thinking skills, including curiosity and scepticism regarding patients as well as clearer communication, significantly influence clinical judgement. This study also highlighted the importance of understanding the subject [35, 36]. Therefore, midwives must recognise minor changes in women’s conditions and determine the best course of action for their care. This is especially important in in-hospital midwife-led births, in which the midwives are the primary caregivers in charge of the birth. Midwives must be able to detect any changes in women’s condition early to prevent them from experiencing complications [14, 37]. They need to develop concern for women, recognise changes in their condition, and gather information regarding their circumstances during conversations.

### Determining the best care while engaging in thorough reasoning

Midwives who maintain close and continuous support for labouring women are particularly well positioned to detect subtle changes in these women’s condition [38]. They not only observe data from the cardiotocogram and other sources but also gather information by observing women’s facial expressions and listening to their words. Midwives engage in clinical reasoning by synthesising and interpreting this information [39]. A large amount of reasoning is significant in determining the best care for women based on several hypotheses. When their clinical reasoning is limited, midwives may struggle to consider a woman’s condition from multiple perspectives, potentially leading to a narrow or incomplete understanding of her needs. Skilled midwives can filter relevant information from a large volume of data, relate it effectively, and deepen their understanding of women’s condition through iterative reasoning processes [32]. A midwife’s ability to engage in complex and varied reasoning is crucial for determining the most appropriate care for women. This is insufficient for midwives to understand women’s condition. They must provide predictive care to prevent women from deviating from normality [40]. This requires education that fosters thinking skills so that midwives can apply more reasoning.

### Adjusting the practice based on the needs and circumstances of labouring women

Labouring women deserve the highest quality of care, particularly given the dynamic nature of birth, during which their condition can change rapidly. Midwives make care decisions based on their reasoning; however, this may not always be the most appropriate approach. When a planned intervention proves ineffective, midwives promptly reassess the situation and determine the next-best course of action. In this way, midwives quickly evaluate care interventions based on the responses of labouring women [41]. In clinical judgement, reflecting on practice is crucial. By utilising past experience to evaluate this practice, future performance can be enhanced [35]. Midwives can adjust the direction of care as needed, enabling women to experience a normal process and deliver their babies safely. To do so effectively, midwives must demonstrate both resourcefulness in adapting their reasoning to each woman’s unique circumstances and patience in providing continuous, attentive care [42]. Midwives who are engaged in in-hospital midwife-led births have a sense of mission to safely guide labouring women to a natural birth because of the absence of an obstetrician, which we speculate may influence the midwives’ initiative [43].

### Strengths and limitations

This study had several notable strengths. The primary strength was the real-time elicitation of midwives’ clinical judgement. Unlike previous studies that relied on retrospective accounts of past experiences, this study captured midwives’ reasoning as it occurred in practice. Because clinical judgements are made instantaneously in real time, collecting verbalisations in real time allowed for the capture of more accurate and authentic data, thereby minimising recall bias. Second, the childbirth process was continuously observed from admission to delivery with participant observation data supplemented by video recordings. This ongoing videotaping enabled us to monitor the condition of the women before any judgements were made. As clinical judgements are typically based on available information, collecting objective data on the women’s condition is essential. In this study, we analysed both information regarding women’s condition and the clinical judgements made. This allowed us to provide a detailed account of the circumstances under which these clinical judgements were made.

This study had several limitations. One notable limitation was the number of sites and hospital size. This study was conducted at three hospitals, one of which managed high-risk births. Midwives at this hospital were responsible for both midwife- and physician-led high-risk births, which may have provided them with a broader perspective on regularly detecting abnormalities. In the future, we can identify areas in which midwives need further training by examining the differences in clinical judgement skills among midwives working in facilities that focus solely on in-hospital midwifery compared to those that manage high-risk deliveries alongside in-hospital midwifery.

## Conclusion

We were able to elucidate the detailed clinical judgement process by employing Tanner’s model framework and conducting a deductive analysis to clarify the midwives’ clinical judgement process. We identified seven categories as characteristic clinical judgement processes for midwives engaged in in-hospital midwife-led births during delivery: ‘Predicting the condition of labouring women and determining the initial direction of care upon taking responsibility’, ‘Gathering information by asking questions in different ways to identify minor changes in labouring women’s condition’, ‘Examining the cause of the problem from available information to uncover and determine the root cause’, ‘Predicting birth progress and determining care based on information beyond internal examinations’, ‘Instantly modifying the direction of care based on labouring women’s condition’, ‘Determining what care should be provided immediately based on predictions of birth progression’, and ‘Providing continuous care based on labouring women’s responses to care’. These findings demonstrated the high level of clinical judgment possessed by midwives engaged in in-hospital midwife-led births, and suggest that this high level of judgment guides women toward safe and highly satisfying childbirth experiences. Midwives involved in in-hospital midwife-led births predicted their patients’ future course of events from the moment they took charge. They remained highly attentive to even the slightest changes during the labour process and integrated information gathered through their five senses to assess the progression of delivery and provide care. The midwives adapted their approach based on women’s responses, consistently ensuring the best possible care throughout the process. The ability to provide personalised care is a distinctive strength of midwives stemming from their close connection with the labouring women in their care. Midwives need to foster the ability to accurately and comprehensively assess labouring women using various sources of information and to develop critical thinking skills that allow them to anticipate future developments.

## Data Availability

No datasets were generated or analysed during the current study.

## Abbreviations

ROP: Right occiput posterior
CTG: Cardiotocogram
